# Morphology and thickness of the buccal bone wall of the maxillary central incisors in population: a CBCT study

**DOI:** 10.3389/fdmed.2024.1472028

**Published:** 2024-11-29

**Authors:** Yaping Song, Song Yang, Chao Wang

**Affiliations:** ^1^ The Affiliated Traditional Chinese Medicine Hospital, Guangzhou Medical University, Guangzhou, China; ^2^ Fujian Key Laboratory of Oral Diseases, Fujian Provincial Engineering Research Center of Oral Biomaterial and Stomatological Key Laboratory of Fujian College and University, Fuzhou, China; ^3^ Department of Prosthodontics, School and Hospital of Stomatology, Fujian Medical University, Fuzhou, China; ^4^ Hospital of Stomatology, Guanghua School of Stomatology, Sun Yat-sen University, Guangzhou, China

**Keywords:** maxillary central incisor, buccal bone wall, anatomical classification, immediate implant, CBCT

## Abstract

The objective of this study was to measure and analyze the anatomical morphology and thickness of the buccal bone wall (BBW) of the maxillary central incisors, providing a theoretical basis for immediate implant surgery. A total of 372 maxillary central incisors were collected and classified into B and P types based on the root position and the degree of coverage of the BBW. The cases were divided into male and female groups, with 180 males and 192 females. The thickness of the BBW was measured at three measurement locations: 4 mm apical to the cementoenamel junction (CEJ), the mid-root, and the root apex. The number and proportion of various types of BBW are as follows: B1 (54, 14.52%), B2 (72, 19.35%), B3 (61, 16.40%), P1 (76, 20.43%), P2 (66, 17.74%), and P3 (43, 11.56%). In the B type group, the thickest BBW at 4 mm apical to the CEJ and the mid-root was observed in B3 (0.89 mm ± 0.09 mm, 0.56 mm ± 0.07 mm). The thickest BBW at the root apex was observed in B2 (0.46 mm ± 0.05 mm). In the P-type group, the thickest BBW at all three measurement locations was observed in P3 (1.10 mm ± 0.08 mm, 1.04 mm ± 0.11 mm, 3.59 mm ± 0.12 mm). The BBW of the maxillary central incisors in males was thicker than that in females. The conclusion drawn was that most BBW of the maxillary central incisors are thin, with a portion of the maxillary central incisors having only a thin BBW coverage at 4 mm apical to the CEJ and no significant bone wall coverage elsewhere. This Type of maxillary central incisor presents a higher risk of buccal soft and hard tissue recession and even bone fenestration after implant surgery. It is, therefore, crucial to assess the three-dimensional position of the root and measure the thickness of the BBW using Cone-beam computed tomography (CBCT).

## 1 Introduction

In recent decades, dental implants have become a reliable treatment for tooth loss. Immediate implant placement, has become the preferred treatment for patients needing upper anterior teeth extraction due to trauma or caries. This approach restores aesthetics and function in a shorter time frame, avoiding the trauma of a second surgery post-extraction and preserving the width and height of the remaining alveolar bone (1). The accurate three-dimensional position of the implant is an absolute prerequisite for immediate implantation in the aesthetic zone. The unpredictability of soft and hard tissue reconstruction during immediate implantation increases the aesthetic risk. It is crucial to determine the implant placement based on the BBW thickness and anatomical morphology after tooth extraction.

Adequate thickness of the BBW is vital for the initial and long-term stability of implants in the anterior maxillary region (2, 3). Most Clinicians agreed that at least 2 mm of bone tissue should be retained on the buccal side of the implant for long-term aesthetic success (4). However, literature reported that the BBW in the population is typically less than 1 mm thick, with nearly half having a thickness of less than 0.5 mm (5, 6). Such thin BBW may increase the likelihood of tissue recession around implants post-surgery (7). Therefore, considering the BBW anatomical morphology of the upper anterior teeth is critical in treatment planning before extraction or implant placement.

Recent clinicians have used CBCT to measure the thickness of the BBW of the maxillary anterior teeth and classified and analyzed the measurement data according to age and gender differences (8, 9). However, in the upper anterior region, in addition to the varying thickness of the BBW, the anatomical morphology of the BBW is also different, significantly impacting the long-term success of implant surgery. Currently, only a few studies have reported on the impact of BBW morphology on immediate implant placement, and the analysis still needs to be improved (10, 11). In this study, the maxillary central incisors of the patients were scanned with Cone-beam computed tomography (CBCT), and the BBW was classified according to the different anatomical morphologies. The BBW thickness was then measured at 4 mm apical to the CEJ, the mid-root, and the root apex of the maxillary central incisor. The measurement results were compared and analyzed according to gender. This study aimed to evaluate the position of maxillary central incisor roots in the alveolar bone and the morphology and thickness of the BBW using CBCT technique. This study expected to provide a theoretical basis for immediate implantation.

## 2 Experimental details

### 2.1 Power analysis

The sample size calculation was performed using the formula $n\;=\;Z^{2}P(1-P)/d^{2}$ (12). Considering a 95% confidence interval (*Z* = 1.96), a 5% precision, and a 50% expected prevalence (maximized due to unpredictability), the minimum number of teeth to be included in the study was determined to be 372.

### 2.2 Study subjects and grouping

The study included 372 maxillary central incisors from patients who received treatment at the Affiliated Traditional Chinese Medicine Hospital of Guangzhou Medical University and Hospital of Stomatology, Sun Yat-sen University, between January 2020 and December 2023 for various reasons requiring CBCT examination. The subjects comprised 180 males and 192 females, aged 20–60 years, with an average age of 35.8 years. There were 200 left maxillary central incisors and 172 right maxillary central incisors.

The inclusion criteria for clinical data were as follows: (a) Chinese population, aged 20–60 years; (b) all maxillary teeth were present, including both left and right sides. The exclusion criteria were images with (a) evidence of tooth trauma or root fracture; (b) CBCT with distorted images or metal artifacts; (c) x-ray images with fillings, restorations, or any evidence of apical lesions, bone loss, or resorption; (d) a history of periodontal and orthodontic treatment; (e) systemic diseases such as diabetes that affect periodontal status. The subjects were divided into two groups based on gender.

### 2.3 Measurement tools and methods

The patients underwent CBCT (New Tom VG, Verona, Italy) scanning in the state of cusp malposition. The head fixation device and cursor positioning system were used to make the midsagittal plane of the subject's face perpendicular to the ground plane, the orbitoauricular plane parallel to the ground plane, the upper and lower teeth kept in the intercuspal position, and the cursor positioning system was aligned with the center of the scanned object, and the upper anterior teeth were scanned. All image data were generated by the same CBCT scan, and the scanning resolution was 0.15 mm. The image analysis was performed on the same computer and the same medical color LCD monitor using the software provided by the CBCT device, and the sagittal continuous tomography observation method of MPR was used to evaluate the labial bone wall thickness of the upper and lower anterior teeth. The image layer thickness and layer spacing were 0.15 mm. Two doctors with rich experience in imaging performed the measurements separately, and 30 cases were randomly selected, and each person was required to repeat the measurement of the same data 1 week later.

### 2.4 Classification of the BBW anatomical morphology

**Figure 1:**
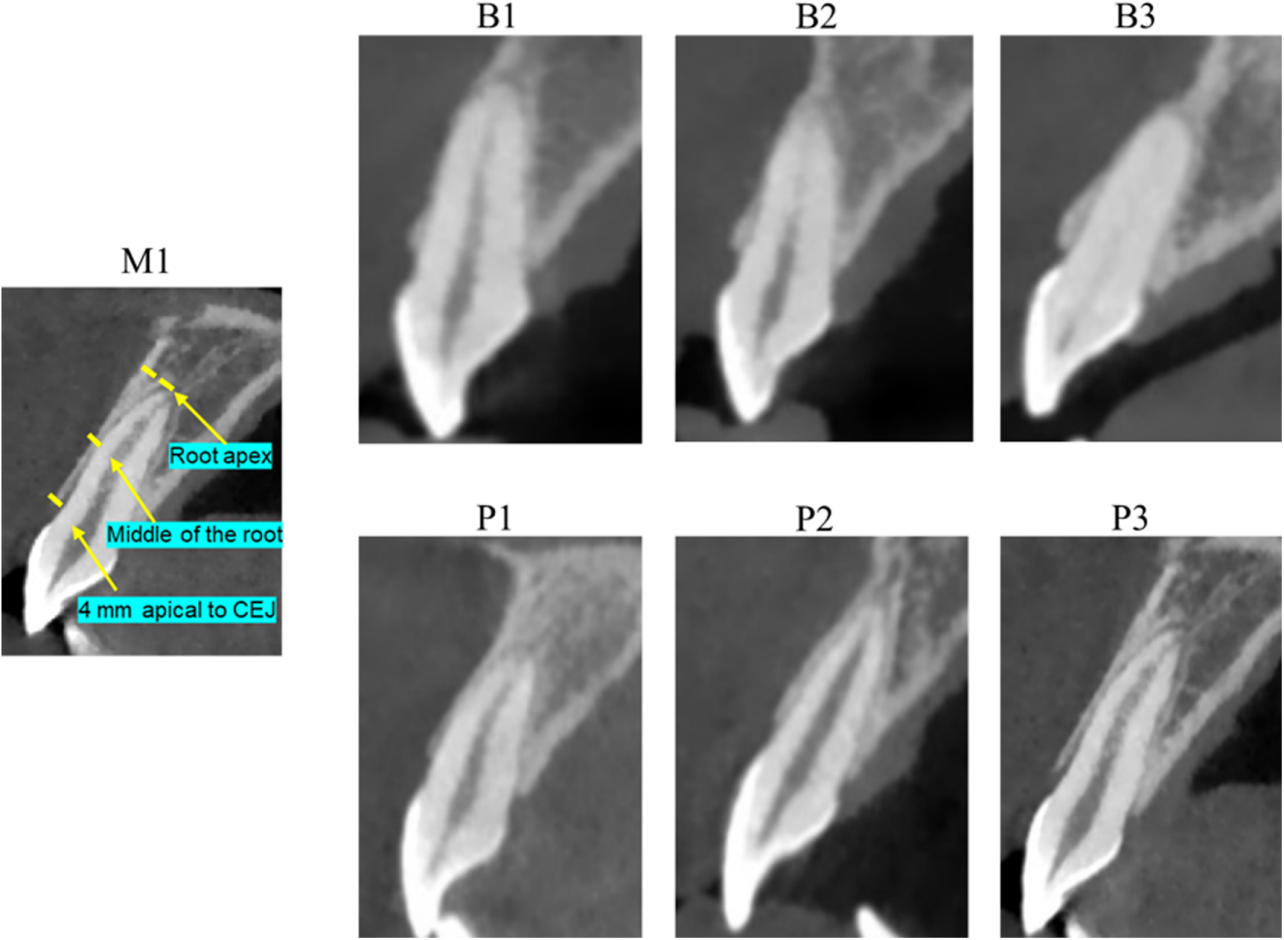
CBCT measurement images of the BBW of maxillary central incisors and CBCT images of various anatomical types of the BBW.

The BBW was classified into B-type and P-type based on its morphology. Each Type was further subdivided into three subtypes according to the thickness of the BBW (Figure 1).

B-type: There is no obvious bone wall coverage or very thin bone wall coverage at the root apex of the maxillary central incisor. There is obvious bone wall coverage only from CEJ to the middle of the root, and the thickness of the bone wall gradually thins toward the root.

B1: Bone wall coverage only at 4 mm apical to CEJ.
B2: Bone wall coverage from CEJ to the middle of the root.
B3: Bone wall coverage from CEJ to the root apex.

P-type: The entire buccal of the maxillary central incisor is covered with bone wall, and the thickness of the BBW is relatively uniform.

P1: Thin bone wall coverage from CEJ to the root, approximately 0.5 mm.
P2: Moderate bone wall thickness from CEJ to the root, approximately 0.5 mm to 1 mm.
P3: Thick bone wall coverage from CEJ to the root, approximately 1 mm.

### 2.5 Measurement content

Measurement location 1: The thickness of the BBW at 4 mm apical to CEJ in the direction perpendicular to the long axis of the tooth.
Measurement location 2: The thickness of the BBW from CEJ to the middle of the root apex in the direction perpendicular to the long axis of the tooth.
Measurement location 3: The thickness of the BBW at the root apex in the direction perpendicular to the long axis of the tooth.

### 2.6 Gender grouping

After classifying the anatomical morphology of the BBW of the maxillary central incisor, the BBW was measured at three measurement locations. The measurement results were grouped by gender and statistically compared to analyze the differences between males and females.

### 2.7 Statistical analysis

SPSS16.0 was used for data analysis and processing, and the *t*-test, mean analysis, and Latin square test were used for comparison between groups. The test level was two-sided *α* = 0.05, and *P* < 0.05 was considered to have a significant difference.

## 3 Results

In the measurement of 372 maxillary central incisors, 100 cases were randomly selected for repeated measurements. The results indicated no significant differences between the two measurements (*P* > 0.05). The thickness of the BBW was measured at three measurement locations: 4 mm apical to the CEJ, the mid-root, and the root apex. The number and proportion of various types of BBW are as follows: B1 (54, 14.52%), B2 (72, 19.35%), B3 (61, 16.40%), P1 (76, 20.43%), P2 (66, 17.74%), and P3 (43, 11.56%). A statistical analysis was conducted to assess the proportion of each anatomical Type according to gender (Table 1).

**Table 1:** Classification of the anatomical morphology of the BBW of the maxillary central incisor (*n*, %).

Gender	Total	B1	B2	B3	P1	P2	P3
All	372	54 (14.52)	72 (19.35)	61 (16.40)	76 (20.43)	66 (17.74)	43 (11.56)
Male	180	27 (15.00)	36 (20.00)	30 (16.67)	35 (19.44)	30 (16.67)	22 (12.22)
Female	192	27 (14.06)	36 (18.75)	31 (16.15)	41 (21.35)	36 (18.75)	21 (10.94)

**Figure 2:**
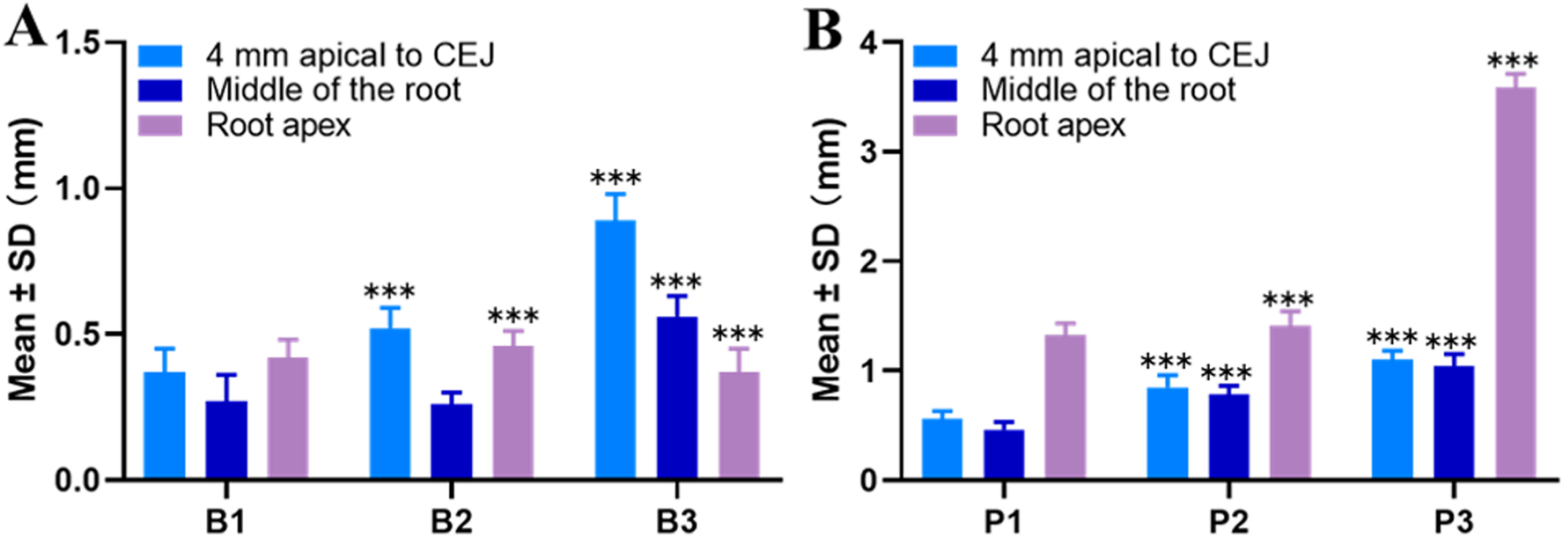
Measurements of the BBW thickness at different measurement locations in maxillary central incisors. **(A)** B-type anatomical morphology; **(B)** P-type anatomical morphology (*P < 0.05, **P < 0.01, ***P < 0.001 compared to the 4 mm apical to CEJ group).

As shown in Figure 2A, in the B-type anatomical morphology of the BBW of the maxillary central incisors, the thickest bone at 4 mm apical to the CEJ and the middle of the root was observed in the B3 type (0.89 mm ± 0.09 mm, 0.56 mm ± 0.07 mm). The thickest BBW at the root apex was observed in the B2 type (0.46 mm ± 0.05 mm). Significant differences were observed in the thickness of the BBW at the three measurement locations (*P* < 0.05). As shown in Figure 2B, in the P-type anatomical morphology of the BBW, the thickest BBW at all three measurement locations was observed in the P3 type (1.10 mm ± 0.08 mm, 1.04 mm ± 0.11 mm, 3.59 mm ± 0.12 mm). Significant differences were observed in the thickness of the BBW at the three measurement locations (*P* < 0.05).

**Figure 3:**
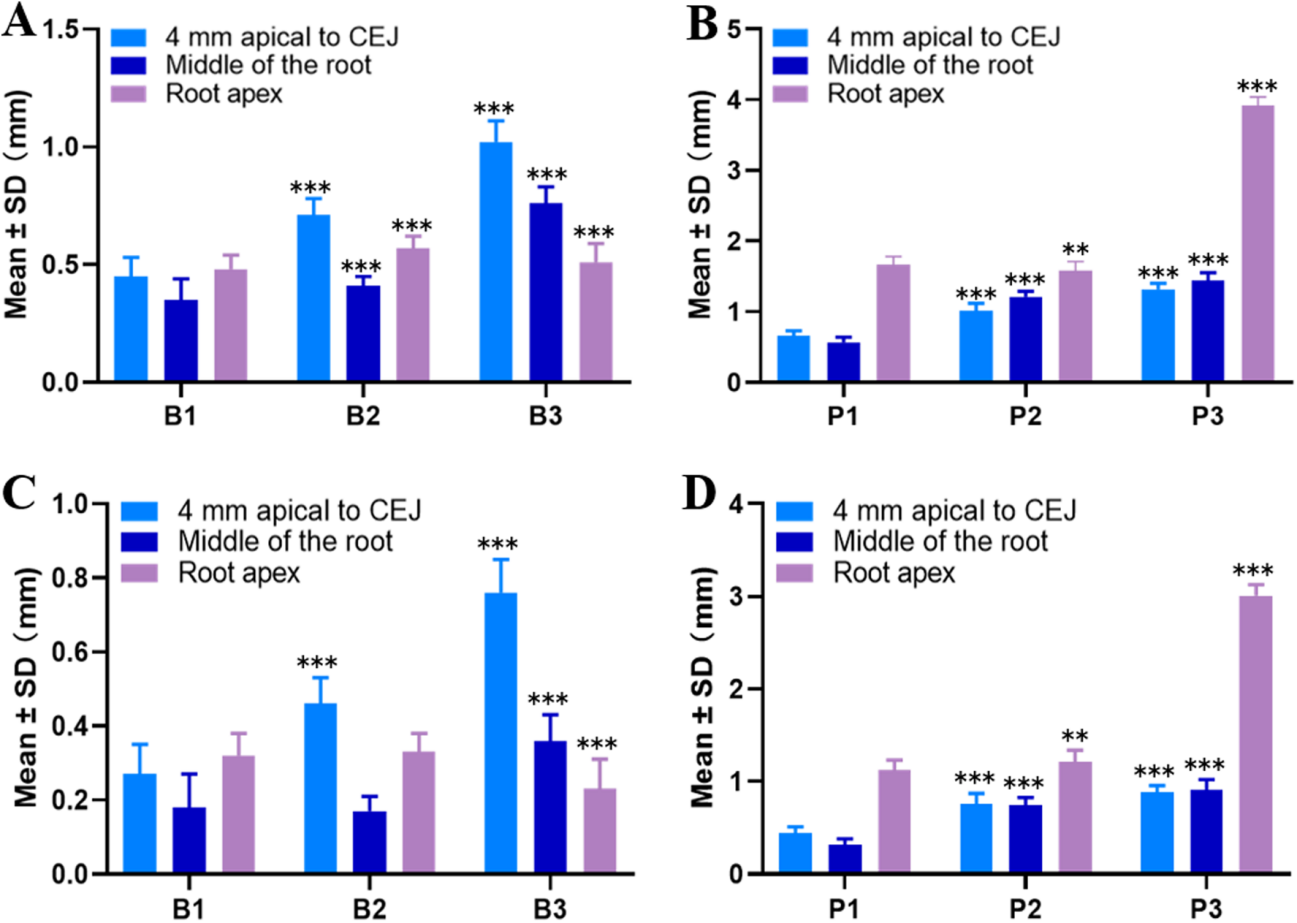
Measurements of the BBW thickness at different measurement locations in maxillary central incisors by gender. **(A)** B-type in males. **(B)** P-type in males. **(C)** B-type in females; **(D)** P-type in females (*P < 0.05, **P < 0.01, ***P < 0.001 compared to the 4 mm apical to CEJ group).

As shown in Figure 3, significant differences in the thickness of the BBW of maxillary central incisors were observed between male and female patients, regardless of B-type or P-type (*P* < 0.05). The BBW thickness in males was thicker than in females at different measurement locations. The thickest BBW was observed in the P3 type in males (1.32 mm ± 0.08 mm, 1.44 mm ± 0.11 mm, 3.92 mm ± 0.12 mm), while the thinnest BBW was observed in the B1 type in females (0.27 mm ± 0.08 mm, 0.18 mm ± 0.09 mm, 0.32 mm ± 0.06 mm).

## 4 Discussions

This study reviewed the anatomical classification and thickness measurements of the BBW of maxillary central incisors in the Chinese population. In the anterior maxillary region, where the BBW is often very thin, the use of CBCT to assess the BBW morphology and thickness before extraction has become a routine diagnostic method. This technique provides technical support to ensure adequate coverage of the BBW during and after implant placement. This study revealed that the BBW was thickest in the P3 type and thinnest in the B1 type, with males generally having thicker BBW than females. These main findings are consistent with other studies and recent literature (13), which have evaluated the anatomical morphology and thickness of the BBW of maxillary anterior teeth and found minimal differences between the Chinese population and those of other nationalities. The thicker BBW in males also aligns with other studies (5, 14). However, some literature presented contrasting views, such as Demircan et al., who found no significant gender differences in BBW thickness in the maxillary anterior region (15). Other studies have reported that the thickness of the BBW is unrelated to gender but is associated with age (16, 17). Age-related factors were not further discussed in this study, primarily because the focus was on the anatomical classification of the BBW while aging increases the likelihood of periodontitis, which introduces more confounding factors. The reason why people over 20 years old were selected as the inclusion criteria in this study is that according to previous literature reports, most patients in the Chinese population only developed all tooth roots well and had stable occlusal relationships around 20 years old (5, 6).

Currently, most studies focus on the anatomical morphology of the entire alveolar bone of the maxillary central incisors (16, 17), with few researchers specializing in the anatomical classification of the BBW. However, the anatomical morphology and thickness of the BBW are critical for immediate implant surgery in the maxillary central incisor region. Kan et al. (18) classified the maxillary anterior teeth into four types based on the inclination angle of the root within the alveolar bone: Type I, II, III, and IV. This method is simple, feasible, and objective, making it easy to summarize and classify. In the sagittal plane, most roots classified as Type I are close to the buccal cortical bone; roots positioned centrally in the alveolar bone, with the apical third not contacting either the buccal or palatal cortical bone, are classified as Type II; roots near the palatal cortical bone are classified as Type III; and those with at least two-thirds of the root length contacting both the buccal and palatal cortical bone are classified as Type IV. Lau et al. divided the position of maxillary central incisors within the alveolar bone into three types: B, P, and M. The B type is defined by the root axis being buccal to the alveolar bone axis, the P-type by the root axis being palatal to the alveolar bone axis, and the M type by the root being between the buccal and palatal bone walls. Lau et al. (19) found that the B type accounted for 78.8%, the M type for 19.4%, and the P-type for 1.8%. This study did not fully adopt either classification method from these two scholars, mainly because the literature review and our preliminary research found that the majority of maxillary central incisors in the population have roots biased toward the buccal side, with significant variation in bone wall coverage at 4 mm apical to the CEJ and the middle of the root (5). Therefore, this study proposed a classification concept specific to the BBW of the population.

The study measured and classified the BBW of the 372 maxillary central incisors. The number and proportion of various types of BBW are as follows: B1 (54, 14.52%), B2 (72, 19.35%), B3 (61, 16.40%), P1 (76, 20.43%), P2 (66, 17.74%), and P3 (43, 11.56%). The similar numbers of B and P types indicate that the BBW of maxillary central incisors in the population is relatively thin. It was found that, except for the root apex of the B3 and P types, the BBW thickness of all other types and regions was less than 1 mm. These findings are consistent with most studies that observed a BBW thickness of less than 1 mm in the anterior maxillary region (20). This result was further confirmed in a clinical study, which found that over 80% of maxillary central incisors had a BBW thickness of less than 1 mm (21). Other studies have reported similar findings, with approximately 76%–89% of the maxillary central incisor region having a BBW thickness of less than 1 mm (22).

In this study, 66.13% of maxillary central incisors (excluding the B1 and B2 types) had relatively thick BBW. Numerous studies have indicated that these types of teeth are generally suitable for immediate implantation, as their thicker and more uniform BBW can maintain the initial stability of the implant (23). The B1 and B2 types, comprising 116 teeth (33.87%), have generally thin BBW, making it difficult to ensure initial stability during implant placement after tooth extraction. For immediate implant surgery in the B1 and B2 types of maxillary central incisor regions, the palatal bone wall must be fully utilized, and the implant insertion angle should be appropriately directed towards the palatal side. Bone augmentation surgery may be necessary before implantation to restore bone width. In this type of patients, the thinner the BBW of the upper anterior teeth is, the higher the risk of gingival recession after immediate implantation. If necessary, subepithelial connective tissue grafts can be used to improve the aesthetic restoration of the upper anterior teeth (24, 25).

This study also analyzed the BBW thickness of maxillary central incisors in male and female patients. Male patients exhibited significantly thicker BBW compared to females, with the thickest BBW being in the male P3 type (1.32 mm ± 0.08 mm, 1.44 mm ± 0.11 mm, 3.92 mm ± 0.12 mm). These results align with the studies by AlTarawneh and AlAli, who also found significant statistical differences between males and females in the BBW thickness of maxillary anterior teeth (26, 27).

**Figure 4:**
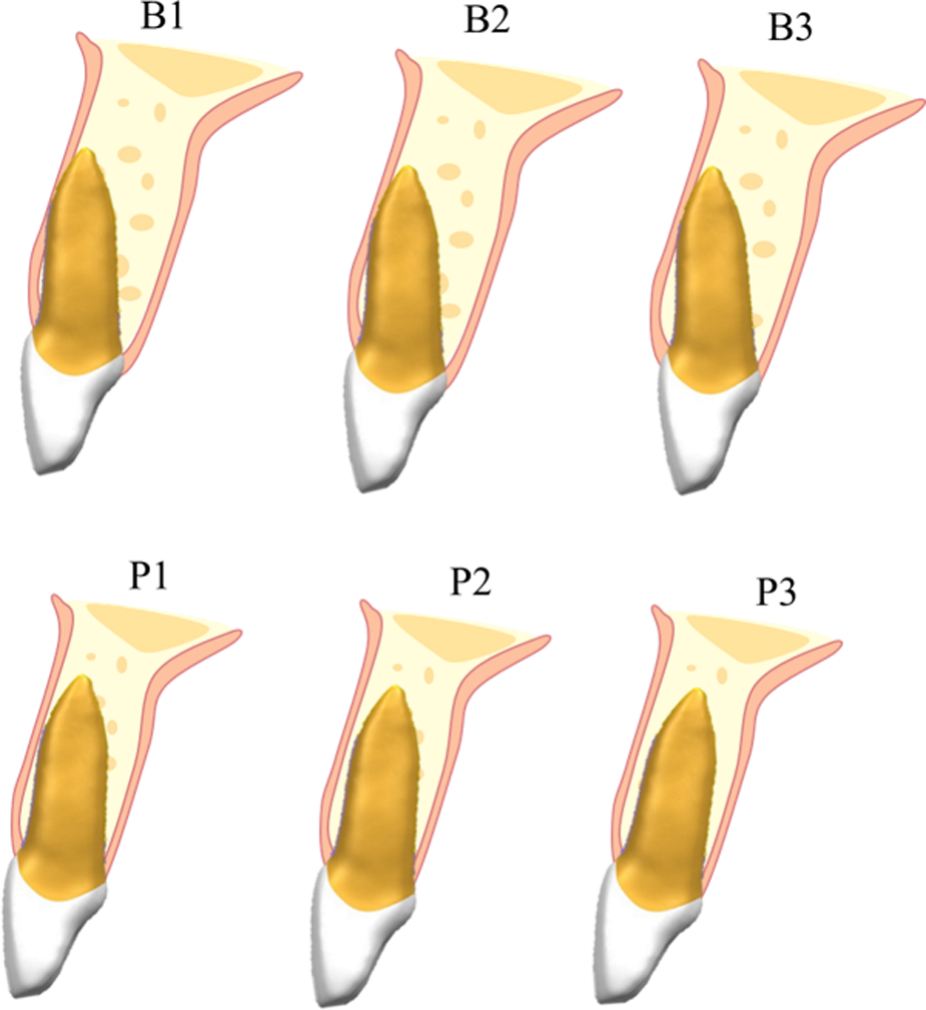
Anatomical classification diagram of the BBW of maxillary central incisors.

In this study, the point 4 mm apical to the CEJ was selected as the measurement location for the BBW of maxillary central incisors (Figure 4). This choice was made because the most likely location for bone fenestration in the anterior maxillary region is approximately 5 mm below the alveolar crest, with minimal bone fenestration occurring above 4 mm apical to the CEJ (28). Elgaddari et al. (29) concluded that the BBW thickness at 4 mm apical to the CEJ could better assess the resorption degree of the BBW after tooth extraction and implantation, as the vertical height of the alveolar bone tends to stabilize approximately 4 mm apical to the CEJ after maxillary central incisors are extracted due to trauma or other reasons. Therefore, measuring the thickness of the buccal and palatal bone walls at 4 mm apical to the CEJ is clinically significant for immediate implant surgery. The mid-root and apical points were selected because these two points are easier to measure clinically, facilitating preoperative measurement and analysis by dentists.

## 5 Conclusion

Most maxillary central incisors have thin BBW, and some maxillary central incisors have only a small amount of bone wall coverage, 4 mm apical to the CEJ, and no obvious bone wall coverage in the rest of the root. These types of maxillary central incisors have a high risk of labial soft and hard tissue recession after implant surgery and may even cause bone fenestration cracking. It is necessary to evaluate the three-dimensional position of the tooth root and measure the BBW thickness through CBCT.

## Data Availability

The original contributions presented in the study are included in the article/Supplementary Material, further inquiries can be directed to the corresponding author.
